# Characterization of human αβTCR repertoire and discovery of D-D fusion in TCRβ chains

**DOI:** 10.1007/s13238-014-0060-1

**Published:** 2014-05-28

**Authors:** Peipei Liu, Di Liu, Xi Yang, Jing Gao, Yan Chen, Xue Xiao, Fei Liu, Jing Zou, Jun Wu, Juncai Ma, Fangqing Zhao, Xuyu Zhou, George F. Gao, Baoli Zhu

**Affiliations:** 1 CAS Key Laboratory of Pathogenic Microbiology and Immunology, Institute of Microbiology, Chinese Academy of Sciences, Beijing, 100101 China; 2 University of Chinese Academy of Sciences, Beijing, 100049 China; 3 Network Information Center, Institute of Microbiology, Chinese Academy of Sciences, Beijing, 100101 China; 4 Chinese Center for Disease Control and Prevention (China CDC), Beijing, 102206 China; 5 Research Network of Immunity and Health (RNIH), Beijing Institutes of Life Science, Chinese Academy of Sciences, Beijing, 100101 China

**Keywords:** TCR repertoire, next-generation sequencing, V/J usage, V-J pairing, CDR3, D-D fusion

## Abstract

The characterization of the human T-cell receptor (TCR) repertoire has made remarkable progress, with most of the work focusing on the TCRβ chains. Here, we analyzed the diversity and complexity of both the TCRα and TCRβ repertoires of three healthy donors. We found that the diversity of the TCRα repertoire is higher than that of the TCRβ repertoire, whereas the usages of the V and J genes tended to be preferential with similar TRAV and TRAJ patterns in all three donors. The V-J pairings, like the V and J gene usages, were slightly preferential. We also found that the TRDV1 gene rearranges with the majority of TRAJ genes, suggesting that TRDV1 is a shared TRAV/DV gene (TRAV42/DV1). Moreover, we uncovered the presence of tandem TRBD (TRB D gene) usage in ~2% of the productive human TCRβ CDR3 sequences.

**Electronic supplementary material:**

The online version of this article (doi:10.1007/s13238-014-0060-1) contains supplementary material, which is available to authorized users.

## INTRODUCTION

The T lymphocytes that are engaged in cellular immunity are mainly αβ T cells. The T-cell receptors (TCRs) located on the T cell surface play key roles in recognition of the vast number of antigenic peptides presented by the major histocompatibility complex (p-MHC). Heterodimeric TCRs are composed of two polypeptide chains, the α and the β chains, each containing one variable domain and one constant domain (Murphy et al., 2007). The antigenic specificity of T lymphocytes is primarily determined by the amino acid sequences of the hypervariable complementarity determining region 3 (CDR3). The nucleotide sequences of CDR3 are generated by somatic recombination of segregated germline variable (V), diversity (D), and joining (J) gene segments for the TCR β chain (TRB), and V and J gene segments for the TCR α chain (TRA).

Aside from αβ T lymphocytes, humans harbor a small subset of T cells called γδ T lymphocytes that possess distinct TCRs on their surface. These TCRs are made up of one γ chain and one δ chain. Resembling the TRB, the nucleotide sequences encoding the TCR δ chain (TRD) variable region are also formed by somatic recombination of the V, D, and J gene segments in the TRD gene locus. This locus is nestled in the TRA locus between the TRAV and TRAJ genes on chromosome 14 (Loh et al., 1988; Takihara et al., 1988). Generally, the TRD genes should only encode TRD (Lefranc, 2001). However, some TRDV gene segments form joints with TRAJ gene and can therefore be used to synthesize TRA. Furthermore, some specific CD8^+^ T cell clones with TRDV1^+^ TRA recognize and kill HIV-infected target cells (Ueno et al., 2003), while other TRDV1^+^ CD8^+^ T cell clones are believed to contribute to chronic neutropenia (Bank et al., 1992; Bank et al., 2003). In total, five out of eight TRDV genes, including TRDV4 to TRDV8, have been classified into this group (Lefranc, 2001), and their names are designated in the form of TRAV/DV plus the corresponding sequential numbers. The remaining TRDV2 and TRDV3 are hypothesized to exclusively rearrange with TRDD genes (Lefranc, 2001). For TRDV1, some reports show that it, resembling the five shared TRAV/DV gene segments, can also rearrange with a small fraction of TRAJ gene segments to synthesize TRA (Miossec, 1990; Miossec et al., 1991).

Somatic recombination begins with lymphoid-specific recognition of conserved recombination signal sequences (RSSs). An RSS that flanks the V, D, and J gene segments consists of a conserved block of seven nucleotides, “the heptamer”, which is always contiguous with the coding sequence, followed by a non-conserved region known as “the spacer”, which is either 12 or 23 bp long, followed by a second conserved block of nine nucleotides, “the nonamer” (Bassing et al., 2002). The recombination occurs between gene segments that are flanked by RSSs of unequal spacer lengths, which is called the 12/23 rule. For immunoglobulin, the D gene segments are flanked by the same RSS containing a 12-bp spacer. It is now confirmed that even though it violates the 12/23 rule, direct joining of one D gene segment to another can occur in most species. In human B cells, D-D fusions are found in approximately 5% of genes that encode antibodies and are the major mechanism accounting for the unusually long CDR3 loops in heavy chains (Larimore et al., 2012; Meek et al., 1989; Murphy et al., 2007). For TRB, inactivation of the TRB enhancer results in non-standard TRBD1-TRBD2 joints in double positive thymocytes in mice (Hempel et al., 1998; Ryu et al., 2003). In humans, the D-D fusions fulfill the 12/23 rule but have only been reported in the TRD (Olaru et al., 2005) and not in the TRB.

Due to the huge diversity of the TCR repertoire, which is estimated to be 2.5 × 10^7^ per individual (Arstila et al., 1999) and theoretically calculated to be much greater (Nikolich-Zugich et al., 2004), it is daunting to precisely and efficiently characterize the repertoire. Classical DNA cloning and Sanger sequencing techniques are laborious and generally have limited throughput (Arstila et al., 1999; Gorski et al., 1994; Pannetier et al., 1993). However, deep sequencing now permits interrogation of complex sequencing targets including the TCR repertoire, at unprecedented depth and reasonable cost (Mardis, 2008; Shendure and Ji, 2008). The diversity, complexity, and specificity of the human TRB repertoire in healthy donors and patients has recently been explored (Klarenbeek et al., 2012; Klarenbeek et al., 2010; Li et al., 2012; Neller et al., 2013; Quigley et al., 2010; Robins et al., 2009; Robins et al., 2010; Sherwood et al., 2011; Venturi et al., 2011; Wang et al., 2010; Warren et al., 2011). However, there is still no comprehensive and systematic study of the TRA repertoire.

Here, we characterized the TRA and TRB repertoires of three healthy donors with distinct human leukocyte antigen (HLA) types and ethnic backgrounds (Table 1). We found that the diversity of the TRA repertoire is higher than that of the TRB repertoire, and the usage of V and J gene segments was preferential for both chains, though the overall frequency pattern was conserved among different individuals. In contrast, for each donor, productive V-J pairings were not equally distributed. Strikingly, the TRDV1 gene was used to the same extent as other normal TRAV gene segments when rearranging with TRAJ segments, indicating that TRDV1 is also a V gene segment shared by the TRA and TRD loci. Furthermore, TRB CDR3 sequence composition analysis uncovered D-D fusions in the TRB, which is likely a key mechanism contributing to the formation of long CDR3 sequences.

**Table 1:** Donor characteristics.

Subject	Sex	Ethnic origin	HLA-A	HLA-B	HLA-Cw
Donor 1	M	Caucasian	*0201/*2501	*0702/*4427	*0501/*0702
Donor 2	F	Mongolian	*0201/*0210	*0801/*4006	*0304/*0702
Donor 3	M	Mongolian	*1101/*2402	*4001/*4001	*0702/*0702

## RESULTS

### Workflow and error correction

We used similar approach as previously reported (Freeman et al., 2009) to profile TCR repertoires derived from 50 mL peripheral blood mononuclear cells (PBMCs). Given that error correction is essential for accurate repertoire profiling, we utilized a series of measures to eliminate errors. First, we used 5′ rapid amplification of cDNA ends (5′RACE) to obtain the TCR V domain transcript sequences. The 5′RACE approach avoids the potential bias associated with the use of the multiple primer sets required to amplify all V region sequences and takes advantage of the conserved sequences offered by TRAC for TRA and TRBC1 and TRBC2 (96% nucleotide sequence identity) for TRB1. Then, two rounds of nested PCR were performed with nested C gene primers to enhance PCR production specificity, ultimately yielding the target sequences with a low level of background amplification. Second, we used high-fidelity reverse transcriptase and DNA polymerase to perform the experiments. For reverse transcription (RT), the reverse transcriptase Superscript II has an error rate of 1/15,000. Subsequently, in the two rounds of PCR, we utilized Plantinum Pfx DNA polymerase with an error rate of 1/632,900. Third, we used the quality score to evaluate sequencing errors. If there were any ambiguous nucleotides within the CDR3 region, that sequence was discarded.

However, for accurate identification of CDR3 sequences, we established bioinformatics tools to specifically deal with the deep sequencing data of the TCR repertoires. To validate the bioinformatics tools, we randomly sampled a subset of the total data and manually dissected the raw reads one by one to identify the CDR3 sequences. This comprised the reference set. The prediction of the reference set by our bioinformatics tools demonstrated an average of 93.02% sensitivity and 98.18% precision (Table 2). We also used the IMGT/HighV-QUEST tool (Giudicelli et al., 2011) to analyze our reference set, and obtained an average of 41.65% sensitivity and 96.15% precision (Table 3). Therefore, our bioinformatics tools can accurately and efficiently identify CDR3 sequences.

**Table 2:** Estimation of the precision and sensitivity of the CDR3-identifying method.

Sample set	No. of reads pairs	Predicted positive segments	True positives	False positives	False negatives	Precision [TP/(TP + FP)]	Sensitivity [TP/(TP + FN)]
TRA-D1*	100,000	8476	8400	478	76	99.10%	94.60%
TRA-D2	10,000	1035	1007	30	28	97.30%	97.10%
TRA-D3	10,000	630	619	69	11	98.30%	90.00%
TRB-D1	10,000	640	625	74	15	97.70%	89.40%
TRB-D2	10,000	880	859	71	21	97.60%	92.40%
TRB-D3	10,000	633	627	36	6	99.10%	94.60%
						98.18%	93.02%

* D1, D2, and D3 represent Donor 1, Donor 2, and Donor 3, respectively

**Table 3:** Estimation of the precision and sensitivity of the IMGT/HighV-QUEST.

Chain Type	No. of reads pairs	Predicted positive segments	True positives	False positives	False negatives	Precision [TP/(TP + FP)]	Sensitivity [TP/(TP + FN)]
TRA	2860	55	53	2	85	96.40%	38.40%
TRB	2610	73	70	3	86	95.90%	44.90%

As shown in Table 4, approximately 60 million 100-nt long paired-end raw reads were generated for each chain from the three donors. Therefore, the quantity of the raw data was kept at a similar level for all three donors. For instance, we identified at least 1.9 million productive TRA CDR3 sequences from Donor 3, with >164,000 unique TRA CDR3 clonotypes. When the flanking V and J gene segments were taken into account, we characterized an average of 2.7 × 10^5^ and 1.6 × 10^5^ distinct TRA and TRB clonotypes, respectively, for all three donors. Notably, we observed that the TRA repertoire was more diverse than the TRB repertoire in all three donors from the sampled PBMCs, regardless of their disparate HLA backgrounds.

**Table 4:** TCR sequence statistics.

Subject	Chain type	Raw reads	Total CDR3 sequence	Unique CDR3 (nt)	Unique CDR3 (aa)	Unique V(D)J TCR sequences (nt)
Donor 1	α	59,845,380	2,488,327	244,524	179,395	300,935
	β	60,088,204	1,913,167	135,614	102,142	154,851
Donor 2	α	57,261,780	2,991,318	258,163	189,457	318,919
	β	56,050,648	2,414,344	158,174	118,560	182,532
Donor 3	α	60,783,874	1,928,533	164,669	122,348	199,577
	β	56,432,586	1,939,500	121,763	90,426	140,270

### TCRα and TCRβ CDR3 distribution

**Figure 1:**
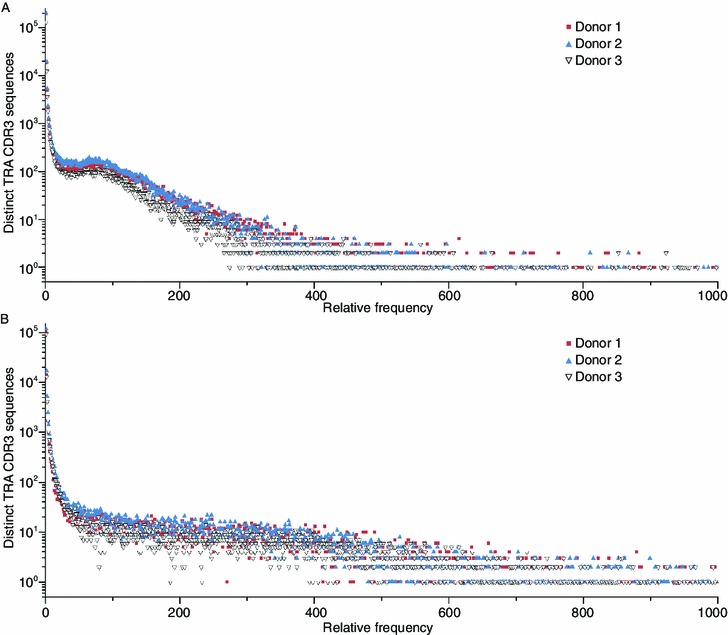
**The distribution of the CDR3 sequences derived from 50 mL PBMC of three donors**. The CDR3 sequence copy number of the TRA (A) and the TRB (B) observed in three donors was represented by the abscissa, while the ordinate represents the number of unique CDR3 clonotypes. For example, the square 300, 10 means that 10 unique CDR3 clonotypes were each observed 300 times. A small set of sequences found with very large copy number (>1000, copies) are not displayed

Figure 1 shows the overall distribution of the TRA and TRB clonotypes and that the abundance of distinct clonotypes can vary several thousand-fold. With respect to the abundance of clonotypes, we classified the TCR clonotypes for both the TRA and TRB repertoires into two groups: group 1 consisted of clonotypes with copy numbers ≤300; and group 2 consisted of clonotypes with copy numbers >300. For group 1, there were 221,835 TRA clonotypes on average (Fig. 1A), which is much greater than that of the TRB clonotypes (i.e., 136,535) (Fig. 1B). For group 2, there were only 616 TRA clonotypes (Fig. 1A) and 1985 TRB clonotypes (Fig. 1B) on average for the three donors.

The characterization of the TRB repertoire has been systematically reported. In this study, we characterized the TRB repertoire in parallel with the TRA repertoire to validate our approaches. As shown in Fig. S1, the TRBV and TRBJ utilization in the productive CDR3 sequences was highly non-uniform in a given individual. Furthermore, inter-donor conservation of TRBV and TRBJ usage (Fig. S1A and S1B, average Pearson correlation coefficients = 0.83 and 0.85) was observed. The TRBV-TRBJ pairings (Fig. S1C) were biased toward specific TRBV-TRBJ pairs, while striking similarity in the pairing frequency among individuals was observed (average Pearson coefficients = 0.65 (*P* < 0.001)). In aggregate, these results are consistent with previous reports (Freeman et al., 2009; Robins et al., 2009; Warren et al., 2011), which validates our approaches.

### TRAV/TRAJ usage and TRAV-TRAJ pairing pattern in healthy donors

**Figure 2:**
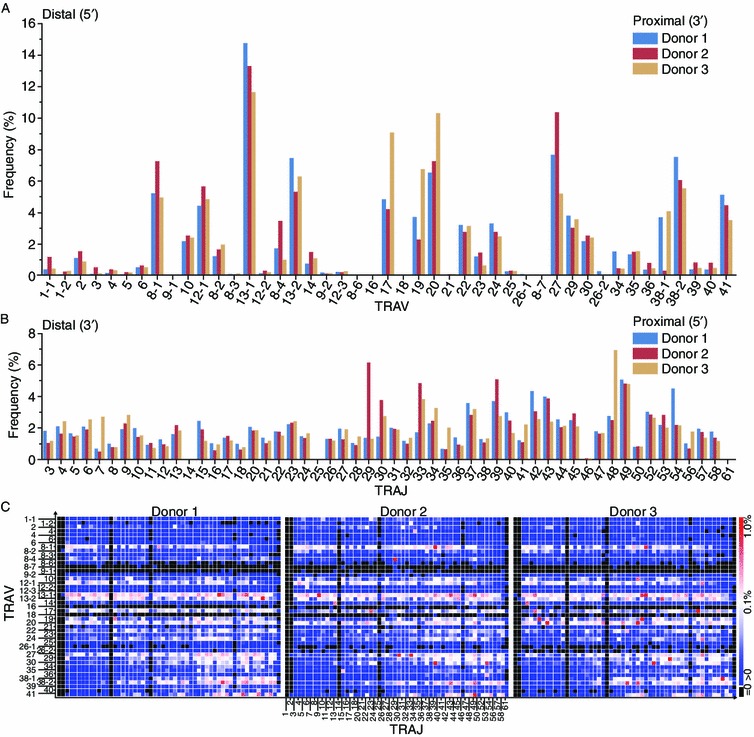
**TRAV, TRAJ gene usage and TRAV-TRAJ pairing are highly correlated among donors**. Relative frequency of TRAV and TRAJ segments are listed in (A) and (B), respectively, according to their chromosome locations. (C) The heat map of the TRAV and TRAJ pairings for three donors are shown

When examining the frequency of the TRAV segments listed according to their chromosomal locations, we found that the TRAV usage was notably biased in a given individual (Fig. 2A). Some TRAV segments such as TRAV8-1, 13-1, 20, 27, and 38-2 are preferentially used in comparison with those like 8-3, 8-6, and 8-7, which are almost undetectable. Furthermore, the frequencies of the TRAV segments that are most proximal to the TRAJ cluster were not the highest, while those most distal to the TRAJ cluster were not the rarest. These data indicate that TRAV segments were selected irrespective of distance from TRAJ gene segments. Most intriguingly, pairwise comparisons of TRAV usage between donors produced a Pearson correlation coefficient of 0.90 ± 0.04 (mean ± SD), indicating marked similarity in the TRAV frequency among individuals.

Likewise, TRAJ usage was also non-uniform in a given donor (Fig. 2B). Furthermore, the TRAJ usage patterns observed in the three healthy donors were quantitatively similar to each other, with an average Pearson correlation coefficient of 0.66 ± 0.04 (mean ± SD).

As illustrated in the heat map (Fig. 2C), the abundance of TRAV-TRAJ pairings was strongly correlated among individuals. The average Pearson coefficient of TRAV-TRAJ pairing was 0.334 (*P* < 0.001). Though the TRAV and TRAJ gene segments usage was strikingly quantitatively similar among donors, the extent of TRAV-TRAJ pairing similarity was somewhat reduced. Especially when focusing on the most abundant TRAV-TRAJ parings, we found that they were unique for each individual. Moreover, as the TRAV and TRAJ gene segments are shown according to their chromosomal positions (5′ to 3′ direction), we determined that the TRAV-TRAJ pairing in humans is not compatible with the sequential coordinate gene recombination hypothesis, which means 5′ to 3′ polarized utilization of the TRAJ library may be coordinated with a 3′ to 5′ polarized utilization of the library of the TRAV gene segments (Fuschiotti et al., 2007; Huang and Kanagawa, 2001; Krangel, 2009; Pasqual et al., 2002; Roth et al., 1991). This situation is analogous to a recent report focusing on TRA in mice (Genolet et al., 2012).

### TRDV1 is used as a common TRAV gene segment: TRAV42/DV1

**Figure 3:**
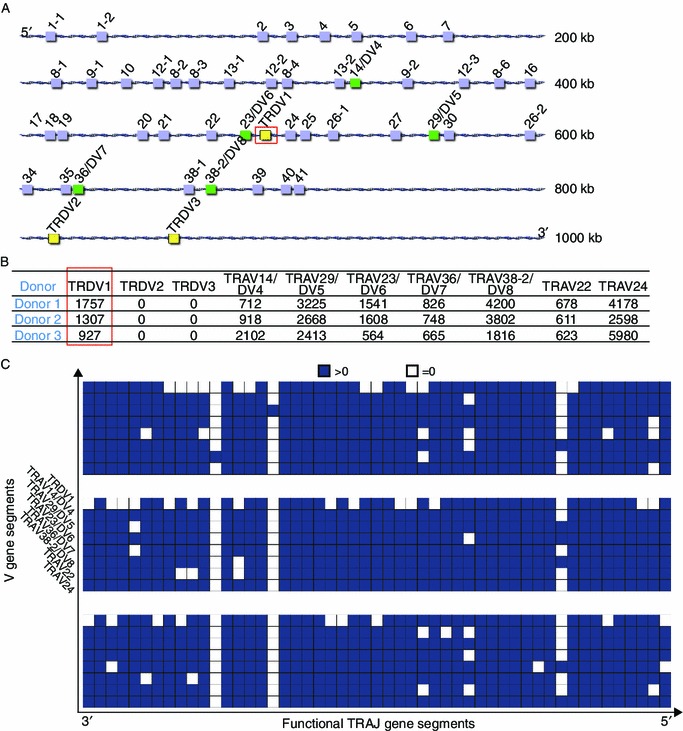
**TRDV1 is also a shared TRAV/DV gene segments**. (A) TRDV gene segments dispersed in the TRA locus in chromosome 14 (14q11.2). TRDV1, TRDV2, and TRDV3 (yellow squares) were not shared with TRA in previous report, while the other five TRDV segments (green squares) also have their corresponding TRAV names. (B) Reads containing TRDV gene segments and two TRAV segments closest to TRDV1 in chromosome were identified. The counts of the reads were listed. (C) The patterns of related TRAJ segments paired with TRDV1 in three donors were shown. The TRAJ gene segments were listed along 3′ to 5′ direction in the chromosome

The TRD locus spans 60 kb on chromosome 14 at 14q11.2 and is nested within the TRA locus (Fig. 3A). The TRD locus is composed of a cluster of one TRDV gene (TRDV2), three TRDD genes, and four TRDJ genes, upstream of the unique TRDC gene (Lefranc, 2001). Another TRDV gene (TRDV3) is located downstream of the TRDC gene, in inverted transcription orientation (Lefranc and Rabbitts, 1990). Resembling the five TRAV/DV gene segments, TRDV1 is dispersed in the TRAV cluster rather than TRDV2 and TRDV3 (Lefranc, 2001). Hence, TRDV1 has a high potential to be a shared TRAV/DV segment.

As illustrated in Fig. 3B, TRDV1 had at least 927 copies, while both TRDV2 and TRDV3, which are believed to exclusively join to TRDD segments (Lefranc, 2001), had zero copies in all donors after noisy sequence elimination. Furthermore, TRDV1 had the same usage level as the other five shared TRAV/DV segments, for which the number of copies ranged from several hundred to several thousands (Fig. 3B).

The TRA gene locus contains 61 TRAJ gene segments, of which 50 are functional, while the others are pseudogenes or open reading frames (ORFs). We found that there was absolutely no signal from the pseudogenes and ORFs in all three donors. For simplicity, we only present the data for genes referred to as functional in the IMGT database. In fact, the rearrangement of TRDV1 with TRAJ is comparable with that of all of the other TRAVs (Fig. 3C). For the other five shared AV/DV segments referred to above, conserved results were also observed for their V-J pairing pattern (Fig. 3C).

The frequency of specific TRDV1-TRAJ recombination within any given donor varied approximately 1000-fold (Table S1). Pairwise comparisons of TRDV1-TRAJ pairings between donors produced an average Pearson correlation coefficient of 0.99 for all three donors, demonstrating that the TRDV1-TRAJ pairing patterns are constant among donors.

These results on TRDV1 usage and TRDV1-TRAJ pairing patterns demonstrated that TRDV1 is also used as a normal TRAV gene segment. With 75% identity at the nucleotide level as the threshold of being a subgroup for the gene segments (Lefranc, 2011), we designated TRAV42/DV1 as the new name for TRDV1.

### Shared CDR3 sequences of TRA are the dominant species

**Figure 4:**
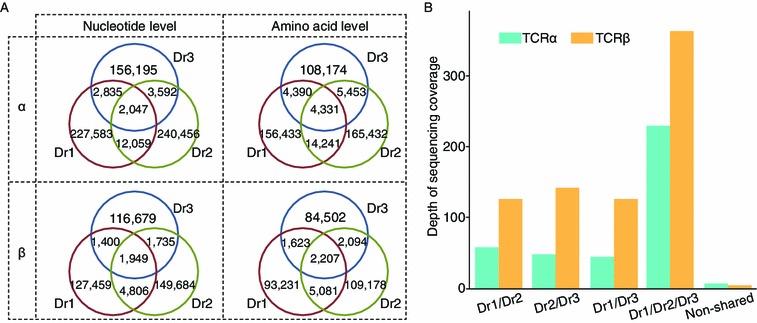
**Shared CDR3 sequences are dominant species**. (A) For TRA and TRB, the number of nucleotide and amino acid clonotypes of CDR3 sequences that are shared by two or three donors are illustrated in a pie. (B) Comparison of the average depth of the shared CDR3 sequences with non-shared ones. Dr1, Dr2, and Dr3 represent Donor 1, 2 and 3 respectively

To compare repertoire diversity among the donors, we obtained the same amount of PBMCs from all three with informed consent. Furthermore, the amount of mRNA used as the template for all three donors in the 5′RACE reactions was kept stringently the same. In this manner, we could accurately compare the repertoire among donors. As shown in Fig. 4A, at the nucleotide level, each repertoire was mostly unique. Donor 1 was a Caucasian, and the other two donors were Mongolian. Donor 1 shared a higher HLA type similarity with Donor 2 than with Donor 3 (Table 1). Donor 1 shared 5.8% of his sampled TRA CDR3 sequences with Donor 2 but only 2% with Donor 3. We also performed a similar analysis based on the translated CDR3 sequences, where Donor 1 shared 10.4% and 4.7% of his sampled TRA CDR3 sequences with Donors 2 and 3, respectively. This is consistent with the common concept that there are preferred amino acid sequences reactive with any given antigenic epitope. Furthermore, these preferred amino acid sequences are coded by a larger diversity of nucleotide sequences due to the degenerated genetic code. In addition, the patterns of sharing for CDR3 sequences of TRB were similar to that of the TRA CDR3 sequences between the three donors (Fig. 4A).

We next classified sequences into shared subsets versus unique subsets for a given donor. The TRA CDR3 sequences shared by two donors had an average depth of 52, which means that any TRA CDR3 clonotype in the shared subsets was present in 52 copies on average (Fig. 4B). The TRA CDR3 clonotypes shared by all three donors had a deeper depth of 230, while the unique clonotypes only had an average depth of 7. This demonstrated that shared TRA CDR3 sequences are usually more abundant than unique clonotypes, and the more people that share the sequences, the more highly abundant they are. This is consistent with the notion that infection with common public pathogens can result in dominant public TCR clonotypes. Our data also demonstrate the consistent sharing pattern of the CDR3 sequences in the TRB repertoire (Fig. 4B).

### D-D fusion is found in TRB

While dealing with the CDR3 sequence dataset, we found the existence of long CDR3 sequences (longer than the average 15 amino acids) in the TRB repertoire. We dissected these longer CDR3 sequences to investigate their composition. There are two TRBD gene segments involved in TRB somatic recombination. Due to nucleotide nibbling at both ends of D gene segments during recombination, TRBD gene identification is challenging for a mature CDR3 sequence. Here, we used more stringent criteria than the Junction Analysis tool (which is specially designed to annotate the TCR CDR3 sequences by IMGT) to identify the TRBD gene segments. We restricted our analysis to include only those sequences with: i) at least six nucleotides identical to the intact germline TRBD1 or TRBD2 sequence; and ii) two continuous nucleotides at each ends matching the known germline TRBD gene segments (see MATERIAL AND METHODS). Junction Analysis requires that only two nucleotides of the TRBD should be compared with the user sequences (Yousfi Monod et al., 2004). Not surprisingly, we found tandem D gene usage in some of these long CDR3 segments.

**Figure 5:**
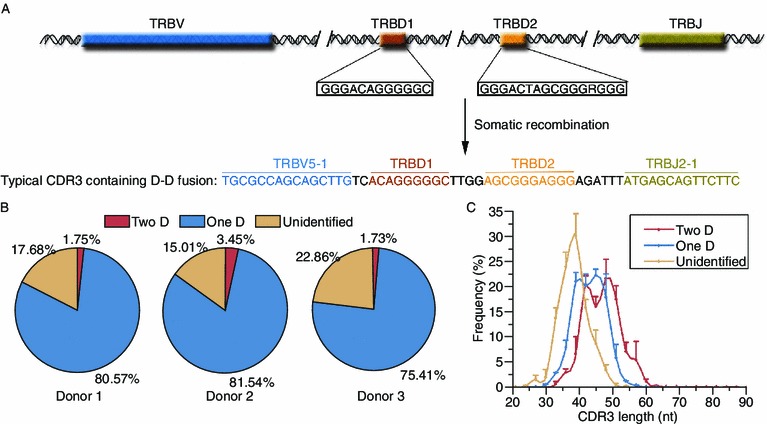
**D-D fusions are found in TRB in human beings**. (A) A typical CDR3 sequence containing D-D fusion was shown. (B) The percentage of the productive CDR3 sequences containing D-D fusions in three donors. (C) The CDR3 length distributions for the productive TRB CDR3 containing two D segments, one D segment and no D segment. The mean frequency of the three donors is shown, with standard deviations as error bars

We randomly select an 18-amino acid-long raw read, which had been identified as containing D-D fusions by our approach, and then analyzed it with the IMGT/Junction Analysis program. The program delimited the CDR3 sequence and showed all of the potential rearranged TRBD gene segments (Fig. 5A). Both TRBD1 and TRBD2 were identified with high scores (nine and 10 nucleotides were identical to germline TRBD1 and TRBD2, respectively). We also collected a panel of reads containing D-D fusions in our dataset and placed them in the supplementary material.

As shown in Fig. 5B, 1.75%, 3.45%, and 1.73% of the productive CDR3 clonotypes were identified to contain D-D fusions in Donors 1, 2, and 3, respectively. However, we could not identify any D gene sequences in 17.68%, 15.01%, and 22.86% of the productive CDR3 clonotypes in Donors 1, 2, and 3, respectively. This may be the result of the very short D sequences produced by nucleotide trimming during somatic recombination. Furthermore, the fit of the length distribution of the CDR3 sequences with D-D fusions has a right shift in the abscissa compared to that of the length distribution of the CDR3 sequences with only one D gene segment (Fig. 5C), and those CDR3 sequences in which TRBD could not unambiguously be identified are shortest in average. These results suggest that D-D fusion was a key mechanism that preferentially contributed to the development of the long TRB CDR3 loops.

**Figure 6:**
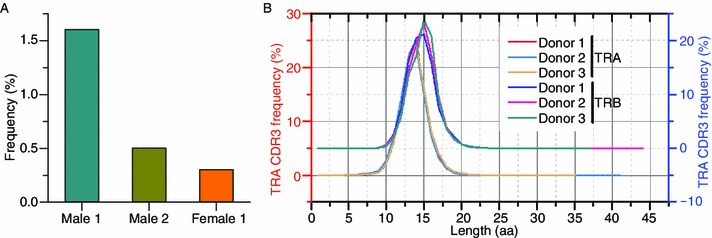
**The confirmation of D-D fusion in the TRB**. (A) Proportion of the TRB CDR3 clonotypes containing D-D fusions from the data published by Warren and his coworkers (Warren et al., 2011). (B) The lengths of CDR3s in both TRA and TRB sequences appear a Gaussian distribution. CDR3 length distribution curves of TRA for three donors all peak at 14 amino acids while that of TRB peak at 15 amino acids

To confirm our findings, we randomly selected approximately 10% of the raw reads published by Holt’s group (Warren et al., 2011) to annotate their CDR3 sequences using our bioinformatics pipeline. As illustrated in Fig. 6A, we also identified 1.6%, 0.5%, and 0.3% of their CDR3 clonotypes containing D-D fusions for their three samples. Furthermore, we compared the CDR3 length distribution of the TRA with that of the TRB (Fig. 6B). The CDR3 length appears as a conserved Gaussian distribution for all three individuals, and the TRA CDR3 sequences peak at 14 amino acids in length. The TRB CDR3 length peaks at 15 amino acids. This is consistent with a previous report (Freeman et al., 2009), which provides further proof that our approaches are reliable. Notably, the TRB CDR3 curve has an appreciable right shift along the abscissa compared to the TRA CDR3 curve for any given donor. The curve shift should be explained by both the D genes and the D-D fusions in the TRB.

In addition, we also analyzed the length distribution and nucleotide composition of the rearranged TRBD segments. The length of rearranged TRBD1 gene segments peak at nine nucleotides (Fig. S2A), while that of TRBD2 peaks at 13 nucleotides (Fig. S2B), implying that the total number of nucleotides trimmed from two ends of TRBD1 and TRBD2 is three on average. All of the productive TRB CDR3 sequences were used to create nucleotide sequence logos (Fig. S2C and 2D). The logos are a graphical representation of a nucleic acid multiple sequence alignment. We observed that the center of the TRBD is more conserved than the flanking regions. This could be explained by nucleotide nibbling (Murphy et al. 2007), though the bias for calling TRBD gene segments cannot be fully ruled out. Regardless, this is consistent with previous reports (Freeman et al., 2009; Quigley et al., 2010).

## DISCUSSION

We used experimental approaches similar to those developed by Freeman and coworkers, and the bioinformatics pipeline we adopted proved to be efficient and accurate in identifying CDR3 sequences from TCR repertoire datasets generated by deep sequencing (Tables 2 and 3). Furthermore, our data on TRB repertoires, including V/J usage, V-J pairing, and CDR3 sharing pattern, is consistent with previous reports. In aggregate, using a similar approach, we effectively identified the TRA CDR3 sequences.

The characteristics of the TRA repertoire are similar to those of the TRB repertoire, indicating that both TRA and TRB experienced similar rearrangement processes regulated by recombination or selection mechanisms. Though the TRA repertoire was estimated to be less diversified than the TCRβ repertoire in whole blood (Arstila et al., 1999), we observed a higher diversity in the TRA repertoire than in the TRB repertoire within the sampled 50 mL PBMCs for any given donor (Table 1). This is consistent with the fact that during the period of 5 days between TRB and TRA rearrangement in the thymus, T cell proliferation results in a 1000-fold expansion, i.e., ~1000 different TRAs may pair with one unique TCRβ (Arstila et al., 1999). Furthermore, dual TCRs caused by less stringent allelic exclusion of the TCRα may also contribute to the TRA-to-TRB pairing (Borgulya et al., 1992; Padovan et al., 1993).

The V-J pairing of each individual displays its own distinguishable traits when focusing on highly abundant pairs. We speculate these differences resulted from different antigen-stimulation backgrounds. For the shared CDR3 sequences, we found that most of them are dominant clonotypes, likely public ones to ward off common pathogens like influenza virus, Epstein-Barr virus, and Cytomegalovirus.

There are contradictory results on the affiliation of TRDV1. Some groups report that TRDV1 can rearrange with some TRAJ gene segments (Bank et al., 1992; Bank et al., 2003; Castelli et al., 1992; Miossec, 1990; Miossec et al., 1991; Ueno et al., 2003), while others believed that it just appears as a normal TRDV gene segment (Lefranc, 2001) that can only be engaged in TRD chain formation. In this study, our approaches could detect transcripts only if they contained the TRAC segment. If TRDV1 can actually rearrange with TRAJ segments, these TRDV1^+^ TRA sequences would also be captured. Indeed, we did find TRDV1^+^ TRA reads in the three donors, and TRDV1 had a similar usage level with shared TRAV/DV segments and the two representative TRAV segments. In addition, nearly all of the TRAJ segments could form joints with TRDV1, which is also solid evidence for TRDV being a shared TRAV/DV gene segment. Interestingly, we noticed that TRDV1-TRAJ20 rearrangement was used 80% of the time. However, whether this is constitutive or antigen-selected is still unknown. The ratio of naïve versus memory T cells deserves more examination.

Somatic recombination in TCR and BCR genes generally fulfills the 12/23 rule (Murphy et al., 2007), but in some cases, it violates this rule. For example, in antibody heavy chain gene recombination, D-D fusions are found in approximately 5% of antibodies (Murphy et al., 2007). The D gene segments of both TRB and TRD are flanked by 12- and 23-bp spacers, and the D-D fusion fulfills the 12/23 rule. The D-D fusion has been identified in nearly all functional TCRδ genes (Olaru et al., 2005), but D-D fusion in TCRβ chains has never been reported (Olaru et al., 2005). While identifying D gene segments, we restricted our analysis to include only those sequences with at least six nucleotides and two or more continuous nucleotides at both ends matching a given germline D gene. At this level of stringency, we identified D-D fusion in ~2% of the productive TCRβ rearrangements. We assume that the actually percentage of D-D fusions should be higher than this because some D gene segments might be excluded by our stringent standards during CDR3 identification. Therefore, 2% is likely an underestimate of the overall rate of human TRB D-D rearrangement.

Larimore and coworkers demonstrate that D-D fusion is a key mechanism resulting in the formation of the long CDRH3 in broadly and potent HIV-1-neutralizing antibodies (bNAbs). They identified D-D rearrangements in ~3% of productive IgH rearrangements. For TCRβ, we obtained similar results, i.e., ~2% of the productive TCRβ were shown to contain D-D fusions. These chains also have a longer CDR3 sequences, but we do not know whether they can recognize non-protein epitopes or they are used to bind inaccessible epitopes like the long CDRH3 in bNAbs. This issue deserves further study. We speculate that the use of two D segments may greatly increase the variability of the β chain because extra N-region nucleotides can be added at the junction between the two D gene segments, as well as at the V-D and D-J junctions. Under these conditions, the potentially huge diversity of the αβTCR repertoire would be further enlarged.

## MATERIALS AND METHODS

### Cell isolation and mRNA extraction

With written informed consent, we draw 50 mL peripheral blood by venipuncture from two males and one female with disparate HLA backgrounds (Table 1). Then 4.7 × 10^7^, 4.6 × 10^7^ and 4.3 × 10^7^ peripheral blood mononuclear cells (PBMC) were isolated immediately by flotation on Ficoll-Hypaque. Using TRIzol (Invitrogen) reagent, the total RNA was extracted from all of the cells according to the manufacturer’s instructions. After that, mRNA was purified from total RNA using the Oligotex mRNA Midi Kit (Qiagen) according to the manufacturer’s specifications.

### 5′RACE

Sequences of variable regions of both TRA and TRB were obtained by 5′RACE, which is consisted of three continuous polymerase chain reactions (PCR), i.e. a reverse transcription PCR (RT-PCR) plus two nested PCRs.

PCRs of α and β chain were performed separately but the conditions were the same. First-strand cDNA was primed using a TCRC gene-specific primer (GSP1), and a target-switching primer (Oligo-dG) was also added as the 5′ template. Each RT-PCR contained 100 ng mRNA, 20 pmol oligonucleotides, 40 nmol DTT, 20 nmol each dNTP, 0.5 μmol Tris-HCl pH 8.3, 0.7 μmol KCl, 30 nmol MgCl_2_ (Invitrogen), and 400 units of Superscript II Reverse Transcriptase (Invitrogen) in a 20-μL volume. Extension was 90 min at 42°C followed by inactivation for 7 min at 72°C. Using an adaptor primer (AP-1) and another C gene specific primer annealing to the 3′ of GSP1, the first nested PCR was carried out with 0.5 μL of the first-strand reactions added as the template. Reaction conditions were as follows: 1 U Platinum Pfx polymerase, 2× Pfx buffer, 1 mmol/L MgSO_4_, primers 0.3 mmol/L each, and 0.3 mmol/L each dNTP in a 50-μL volume. For cycling, a 2 min denaturation at 94°C followed by 30 cycles of 30 s at 94°C, 30 s at 55°C, and 45 s at 68°C, plus a final extension for 5 min at 68°C. The second nested PCR was conducted to enhance the specificity by using another adaptor primer (AP-2) and another gene-specific primer (GSP-3) annealing to a site located upstream of GSP2 in C gene. PCR reaction conditions were the same with the 1st nested PCR except the template was changed into 0.1 μL product of the former nested PCR. Cycling parameters were as follows: a 2 min denaturation at 94°C followed by 20 cycles of 30 s at 94°C and 75 s at 68°C, with an additional extension for 5 min at 68°C. The adaptor primers i.e., the 5′ forward primers in RT, 1st-PCR and 2nd-PCR were depicted earlier (Ozawa et al., 2006). All of the primers were listed in Supplementary Table S1.

### Sonication

Eight aliquots of the 2nd nested PCR reactions were pooled and purified using QIAquick PCR Purification Kit (Qiagen). After 1 h sonication, the eluates were sheared into fragments ranged from 100 bp to 350 bp by bioruptor sonicator (Diagenode). The sample was loaded on an 8% polyacrylamide gel, and the fraction from 150 bp to 300 bp was excised, purified.

### Library construction

The double-stranded DNA fragments were comprised of 3′ or 5′ overhangs. T4 DNA polymerase and Klenow enzyme were then used to convert the overhangs into blunt ends. An ‘A’ base was added to the 3′ end of the blunt phosphorylated DNA fragments, which was ligated with adapters on both ends. The correctly ligated products were purified by agarose gel electrophoresis followed by the QIAquick gel extraction kit (Qiagen). The DNA fragments with adapter molecules on both ends were selected and amplified. The PCR was performed with two primers that anneal to the ends of the adapters. The number of PCR cycles was minimized to avoid skewing the representation of the library. The PCR products were checked and purified by agarose gel electrophoresis. The fragments size and molar concentration of library were respectively determined by the 2100 Bioanalyzer (Agilent) and Real-Time PCR System (ABI).

### Cluster Generation

The Cluster Generation is used to hybridize samples onto a flow cell and amplify them for subsequent sequencing. The major steps include: (1) to hybridize library DNA to flow cell; (2) to extend hybridized templates and perform bridge amplification; (3) to block the flow cell; and (4) to hybridize sequencing primers. The Cluster Generation process was based on Illumina Cluster Station system (cBot), which would automatically amplify a single DNA molecule into clonal clusters for the following sequencing process.

### Sequencing

The sequencing method was based on Illumina HiSeq 2000 platform and it was a fully automated protocol: the clustered template DNA was sequenced by four-color DNA Sequencing-By-Synthesis (SBS) technology with reversible terminators and removable fluorescence. Raw image files were processed by Illumina pipeline for base-calling with default parameters and the sequences of each individual were generated as 100-bp paired-end reads. The sequencing data were subjected to a strict QC test before bioinformatics analysis.

### Identification of full length CDR3 from Assembled reads pair (ARP)

To facilitate the interpretation from tens of millions of reads, we implemented a bioinformatics workflow, including the determination of CDR3, V and J, and tested on human dataset. Manual validation suggests the reliability and implies the applicability for TCR repertoire annotation. (1) Assembled reads pair (ARP) ranges from 140 bp to 174 bp. (2) Built up V peptide dataset from protein sequences of FR3-IMGT region. (3) Built up J peptide dataset from protein sequences of FR4-IMGT region, namely “J^” in our analysis. (4) BLASTx each ARP (with the default parameters) against the above V/J dataset for the finding of FR3-signal and J^-signal.

### Identification of full length CDR3 from raw reads

(1) For 90-bp-long raw reads without assembly, BLASTn (the parameter “word size” was set to 7) for finding V-signal and J-signal by constructing two nucleotide dataset: V-segment dataset comprised all known V exon sequences and J-segment dataset comprised all J exons. (2) Removing false positive reads according to CDR3 length and frame direction.

BLASTx (the parameter “word size” was set to 1) for determining the boundaries of CDR3 by constructing CDR3-V and CDR3-J nucleotide dataset. (1) CDR3-V dataset was extracted from the 6 amino acids in V-segment downstream the conserved “C”; CDR3-J dataset was extracted from 5 amino acids upstream the “FGXG” pattern. (2) In the above two analysis routes, the full-length CDR3 region must be assigned from the conserved “C” to the “F” position of “FGXG” pattern, and have no frameshift and stop codon.

### Combination of the redundant CDR3 segments and V/J determination

(1) Then we combine above results and discard those redundant CDR3 segments according to sequence ID. (2) Quality check of CDR3-containing raw reads. Those sequences with ambiguous nucleotides (i.e. the base-calling result is N) are deleted from the filtered dataset. (3) Determination of the specific V and J segment from CDR3-identified reads.

### D-D fusion identification

First, we identified TRBD1 and TRBD2 from the sequences localized between the TRBV and TRBJ segments. These sequences must be compared to the TRBD1 and TRBD2 gene segments and the alignments with most identical nucleotides are kept as TRBD candidates. If 6 or more nucleotides in a candidate TRBD sequence are identical to the corresponding TRBD segments, it was retained to be analyzed further.

To avoid random matches at both ends of the sequence, 2 or more identical nucleotides with reference sequences at both ends of the candidate TRBD sequences are needed. While TRBD1 and TRBD2 are identified to co-exist in a given CDR3 sequence, and there in no overlap between the two rearranged TRBD segments, the CDR3 was annotated as containing the D-D fusion.

## Electronic supplementary material

Below is the link to the electronic supplementary material.

Supplementary material 1 (PDF 604 kb)

## ABBREVIATIONS

CDR3: complementary determining region 3
RSS: recombination signal sequence
TRA: TCR α chain
TRB: TCR β chain
TRD: TCR δ chain

## References

[CR1] Arstila TP, Casrouge A, Baron V, Even J, Kanellopoulos J, Kourilsky P (1999). A direct estimate of the human alphabeta T cell receptor diversity. Science.

[CR2] Bank I, Book M, Cohen L, Kneller A, Rosental E, Pras M, Bassat IB, Ben-Nun A (1992). Expansion of a unique subpopulation of cytotoxic T cells that express a C alpha V delta 1 T-cell receptor gene in a patient with severe persistent neutropenia. Blood.

[CR3] Bank I, Cohen L, Kneller A, de Rosbo NK, Book M, Ben-Nun A (2003). Aberrant T-Cell Receptor Signalling of Interferon-γ- and Tumour Necrosis Factor-α-Producing Cytotoxic CD8+Vδ1/Vβ16 T Cells in a Patient with Chronic Neutropenia. Scand J Immunol.

[CR4] Bassing CH, Swat W, Alt FW (2002). The mechanism and regulation of chromosomal V(D)J recombination. Cell.

[CR5] Borgulya P, Kishi H, Uematsu Y, von Boehmer H (1992). Exclusion and inclusion of alpha and beta T cell receptor alleles. Cell.

[CR6] Castelli C, Mazzocchi A, Salvi S, Anichini A, Sensi M (1992). Use of the V delta 1 variable region in the functional T-cell receptor alpha chain of a WT31+ cytotoxic T lymphocyte clone which specifically recognizes HLA-A2 molecule. Scand J Immunol.

[CR7] Freeman JD, Warren RL, Webb JR, Nelson BH, Holt RA (2009). Profiling the T-cell receptor beta-chain repertoire by massively parallel sequencing. Genome Res.

[CR8] Fuschiotti P, Pasqual N, Hierle V, Borel E, London J, Marche PN, Jouvin-Marche E (2007). Analysis of the TCR alpha-chain rearrangement profile in human T lymphocytes. Mol Immunol.

[CR9] Genolet R, Stevenson BJ, Farinelli L, Osteras M, Luescher IF (2012). Highly diverse TCRalpha chain repertoire of pre-immune CD8(+) T cells reveals new insights in gene recombination. EMBO J.

[CR10] Gorski J, Yassai M, Zhu X, Kissela B, Kissella B, Keever C, Flomenberg N (1994). Circulating T cell repertoire complexity in normal individuals and bone marrow recipients analyzed by CDR3 size spectratyping. Correlation with immune status. J Immunol.

[CR11] Hempel WM, Stanhope-Baker P, Mathieu N, Huang F, Schlissel MS, Ferrier P (1998). Enhancer control of V(D)Jrecombination at the TCRβ locus: differential effects on DNA cleavage and joining. Genes & Development.

[CR12] Huang C, Kanagawa O (2001). Ordered and coordinated rearrangement of the TCR alpha locus: role of secondary rearrangement in thymic selection. J Immunol.

[CR14] Klarenbeek PL, Tak PP, van Schaik BD, Zwinderman AH, Jakobs ME, Zhang Z, van Kampen AH, van Lier RA, Baas F, de Vries N (2010). Human T-cell memory consists mainly of unexpanded clones. Immunol Lett.

[CR15] Klarenbeek PL, Remmerswaal EB, ten Berge IJ, Doorenspleet ME, van Schaik BD, Esveldt RE, Koch SD, ten Brinke A, van Kampen AH, Bemelman FJ (2012). Deep sequencing of antiviral T-cell responses to HCMV and EBV in humans reveals a stable repertoire that is maintained for many years. PLoS Pathog.

[CR16] Krangel MS (2009). Mechanics of T cell receptor gene rearrangement. Curr Opin Immunol.

[CR17] Larimore K, McCormick MW, Robins HS, Greenberg PD (2012). Shaping of human germline IgH repertoires revealed by deep sequencing. J Immunol.

[CR18] Lefranc MP (2001) Nomenclature of the human T cell receptor genes. Curr Protoc Immunol Appendix 1, Appendix 1O 10.1002/0471142735.ima01os40 18432649

[CR19] Lefranc MP (2011). From IMGT-ONTOLOGY CLASSIFICATION Axiom to IMGT standardized gene and allele nomenclature: for immunoglobulins (IG) and T cell receptors (TR). Cold Spring Harb Protoc.

[CR20] Lefranc MP, Rabbitts TH (1990). Genetic organization of the human T-cell receptor gamma and delta loci. Res Immunol.

[CR21] Li H, Ye C, Ji G, Wu X, Xiang Z, Li Y, Cao Y, Liu X, Douek DC, Price DA (2012). Recombinatorial biases and convergent recombination determine interindividual TCRbeta sharing in murine thymocytes. J Immunol.

[CR22] Loh EY, Cwirla S, Serafini AT, Phillips JH, Lanier LL (1988). Human T-cell-receptor delta chain: genomic organization, diversity, and expression in populations of cells. Proc Natl Acad Sci U S A.

[CR23] Mardis ER (2008). Next-generation DNA sequencing methods. Annu Rev Genomics Hum Genet.

[CR24] Meek KD, Hasemann CA, Capra JD (1989). Novel rearrangements at the immunoglobulin D locus. Inversions and fusions add to IgH somatic diversity. J Exp Med.

[CR25] Miossec C (1990). Further analysis of the T cell receptor gamma/delta+ peripheral lymphocyte subset. The V delta 1 gene segment is expressed with either C alpha or C delta. J Exp Med.

[CR26] Miossec C, Caignard A, Ferradini L, Roman-Roman S, Faure F, Michalaki H, Triebel F, Hercend T (1991). Molecular characterization of human T cell receptor alpha chains including a V delta 1-encoded variable segment. Eur J Immunol.

[CR27] Murphy KM, Travers P, Walport M (2007). Janeway’s Immunobiology.

[CR28] Neller MA, Burrows JM, Rist MJ, Miles JJ, Burrows SR (2013). High frequency of herpesvirus-specific clonotypes in the human T cell repertoire can remain stable over decades with minimal turnover. J Virol.

[CR29] Nikolich-Zugich J, Slifka MK, Messaoudi I (2004). The many important facets of T-cell repertoire diversity. Nat Rev Immunol.

[CR30] Olaru A, Petrie HT, Livak F (2005). Beyond the 12/23 rule of VDJ recombination independent of the Rag proteins. J Immunol.

[CR31] Ozawa T, Kishi H, Muraguchi A (2006). Amplification and analysis of cDNA generated from a single cell by 5′-RACE: application to isolation of antibody heavy and light chain variable gene sequences from single B cells. Biotechniques.

[CR32] Padovan E, Casorati G, Dellabona P, Meyer S, Brockhaus M, Lanzavecchia A (1993). Expression of two T cell receptor alpha chains: dual receptor T cells. Science.

[CR33] Pannetier C, Cochet M, Darche S, Casrouge A, Zoller M, Kourilsky P (1993). The Sizes of the Cdr3 Hypervariable Regions of the Murine T-Cell Receptor Beta-Chains Vary as a Function of the Recombined Germ-Line Segments. Proc Natl Acad Sci USA.

[CR34] Pasqual N, Gallagher M, Aude-Garcia C, Loiodice M, Thuderoz F, Demongeot J, Ceredig R, Marche PN, Jouvin-Marche E (2002). Quantitative and Qualitative Changes in V-J Rearrangements During Mouse Thymocytes Differentiation: Implication For a Limited T Cell Receptor Chain Repertoire. J Exp Med.

[CR35] Quigley MF, Greenaway HY, Venturi V, Lindsay R, Quinn KM, Seder RA, Douek DC, Davenport MP, Price DA (2010). Convergent recombination shapes the clonotypic landscape of the naive T-cell repertoire. Proc Natl Acad Sci U S A.

[CR36] Robins HS, Campregher PV, Srivastava SK, Wacher A, Turtle CJ, Kahsai O, Riddell SR, Warren EH, Carlson CS (2009). Comprehensive assessment of T-cell receptor beta-chain diversity in alphabeta T cells. Blood.

[CR37] Robins HS, Srivastava SK, Campregher PV, Turtle CJ, Andriesen J, Riddell SR, Carlson CS, Warren EH (2010) Overlap and effective size of the human CD8+ T cell receptor repertoire. Sci Transl Med 2, 47ra64 10.1126/scitranslmed.3001442 PMC3212437 20811043

[CR38] Roth ME, Holman PO, Kranz DM (1991). Nonrandom use of J alpha gene segments. Influence of V alpha and J alpha gene location. J Immunol.

[CR39] Ryu CJ, Haines BB, Draganov DD, Kang YH, Whitehurst CE, Schmidt T, Hong HJ, Chen J (2003). The T cell receptor beta enhancer promotes access and pairing of Dbeta and Jbeta gene segments during V(D)J recombination. Proc Natl Acad Sci U S A.

[CR40] Shendure J, Ji H (2008). Next-generation DNA sequencing. Nat Biotechnol.

[CR41] Sherwood AM, Desmarais C, Livingston RJ, Andriesen J, Haussler M, Carlson CS, Robins H (2011). Deep sequencing of the human TCRgamma and TCRbeta repertoires suggests that TCRbeta rearranges after alphabeta and gammadelta T cell commitment. Sci Transl Med 3, 90ra61 10.1126/scitranslmed.3002536 PMC4179204 21734177

[CR42] Takihara Y, Tkachuk D, Michalopoulos E, Champagne E, Reimann J, Minden M, Mak TW (1988). Sequence and organization of the diversity, joining, and constant region genes of the human T-cell delta-chain locus. Proc Natl Acad Sci U S A.

[CR43] Ueno T, Tomiyama H, Fujiwara M, Oka S, Takiguchi M (2003). HLA class I-restricted recognition of an HIV-derived epitope peptide by a human T cell receptor alpha chain having a Vdelta1 variable segment. Eur J Immunol.

[CR44] Venturi V, Quigley MF, Greenaway HY, Ng PC, Ende ZS, McIntosh T, Asher TE, Almeida JR, Levy S, Price DA (2011). A mechanism for TCR sharing between T cell subsets and individuals revealed by pyrosequencing. J Immunol.

[CR45] Wang C, Sanders CM, Yang Q, Schroeder HW, Wang E, Babrzadeh F, Gharizadeh B, Myers RM, Hudson JR, Davis RW (2010). High throughput sequencing reveals a complex pattern of dynamic interrelationships among human T cell subsets. Proc Natl Acad Sci U S A.

[CR46] Warren RL, Freeman JD, Zeng T, Choe G, Munro S, Moore R, Webb JR, Holt RA (2011). Exhaustive T-cell repertoire sequencing of human peripheral blood samples reveals signatures of antigen selection and a directly measured repertoire size of at least 1 million clonotypes. Genome Res.

[CR47] Yousfi Monod M, Giudicelli V, Chaume D, Lefranc MP (2004). IMGT/JunctionAnalysis: the first tool for the analysis of the immunoglobulin and T cell receptor complex V-J and V-D-J JUNCTIONs. Bioinformatics.

