# Supplementary material for: Characterization of human αβTCR repertoire and discovery of D-D fusion in TCRβ chains
Source: Protein Cell. 2014 May 28;5(8):603–15. doi: 10.1007/s13238-014-0060-1 (PMC4130922; doi:10.1007/s13238-014-0060-1)
Supplement: Supplementary file 1 — Supplementary material 1 (PDF 604 kb) [file 13238_2014_60_MOESM1_ESM.pdf]

Supplementary Materials

Figure S1

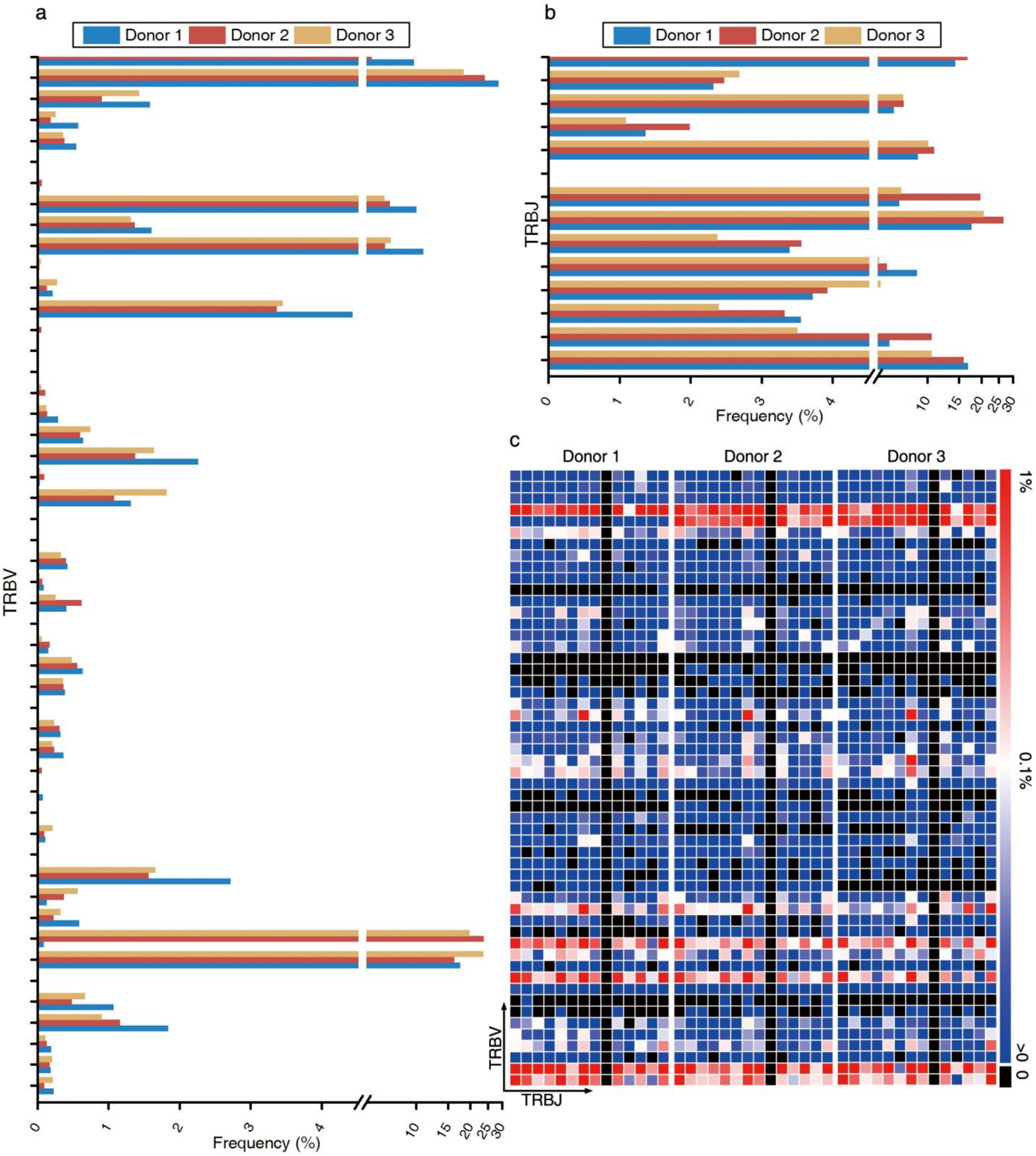

Figure S1 Relative frequency of TRBV and TRBJ segments are listed in (a) and (b), respectively, according to their chromosome locations. (**c**) TRBV and TRBJ pairing

for three donors are shown; similarly, both V and J gene segments are listed in correlation with their chromosomal positions.

Figure S2

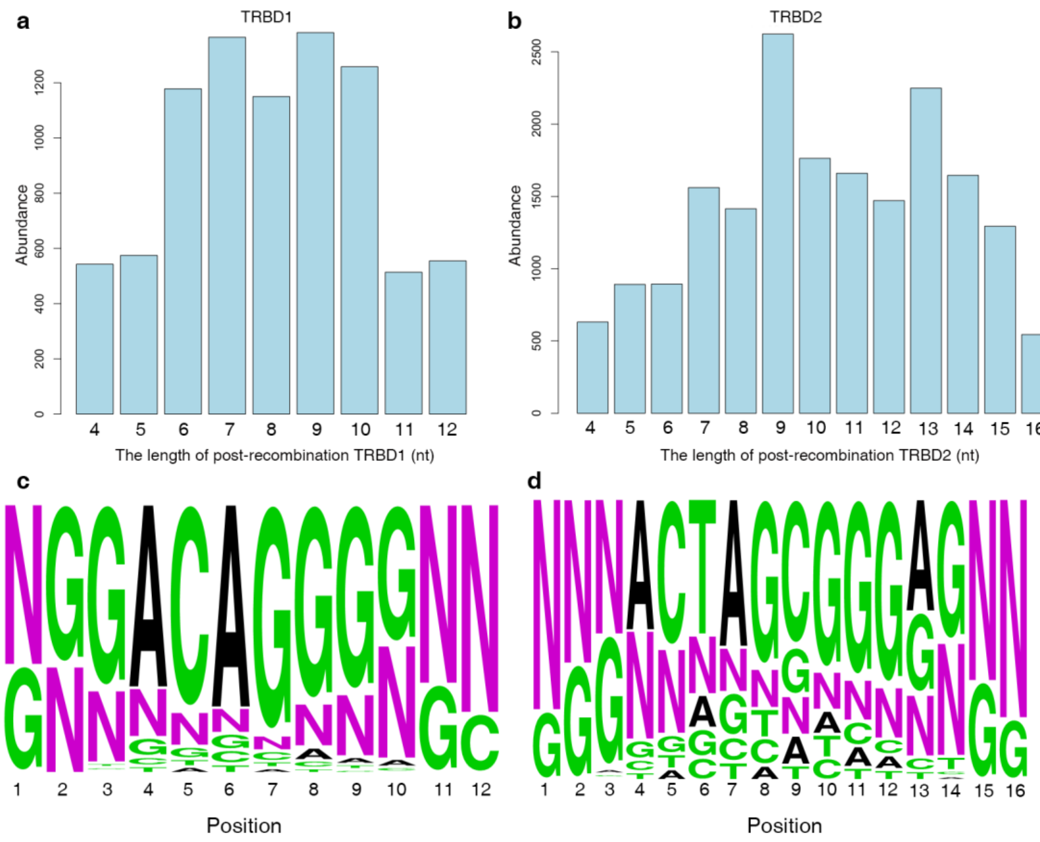

Figure S2 Length distribution and nucleotides compositions of the post-recombination gene segments. TRBD1 (**a**) and TRBD2 (**b**) length distributions are illustrated as columns. Nucleotides compositions are represented using WebLogo for TRBD1 (**c**) and TRBD2 (**d**). N represents the frequency of nucleotides trimmed during somatic recombination.

Table S1 Primers for 5’RACE

Steps	5'primer	3' primer	
		alpha chain	beta chain
RT[a]	ACAGCAGGTCAGTCAAGCAGT AGCAGCAGTTCGATAAGCGGC CGCCATGGAGGGHN	ACACATCAGAAT CCTTACTTTG	GTGGCCAG GCACACCA GTGT
1st PCR	ACAGCAGGTCAGTCAAGCAGT A	GGTGAATAGGCA GACAGACTT	CATTCACC CACCAGCT CAG
2nd PCR	AGCAGTAGCAGCAGTTCGATA A	CACAGCGGCCGC GCTGGTACACGG CAGGGTC	TCTGATGG CTCAAACA CAGC

[a]RT represents Reverse Transcription

Table S2 TRAJ usage in TRDV1^{+} TRA.

	Donor 1	Donor 2	Donor 3
TRAJ3	6	0*	0
TRAJ4	1	2	5
TRAJ5	1	0	0
TRAJ6	2	0	5
TRAJ7	2	0	2
TRAJ8	1	1	1
TRAJ9	2	3	0
TRAJ10	0	0	1
TRAJ11	0	1	0
TRAJ12	0	0	2
TRAJ13	0	20	5
TRAJ15	3	1	1
TRAJ16	0	0	6
TRAJ17	0	1	5
TRAJ18	4	1	14
TRAJ20	1412	1023	766
TRAJ21	20	7	16
TRAJ22	3	6	1
TRAJ23	1	1	6
TRAJ24	1	0	0
TRAJ26	4	2	0
TRAJ27	3	0	3
TRAJ28	0	7	4
TRAJ29	0	2	0
TRAJ30	5	4	3
TRAJ31	5	0	1
TRAJ32	0	0	1
TRAJ33	0	3	3
TRAJ34	3	0	2
TRAJ36	5	6	1
TRAJ37	10	5	1
TRAJ38	1	1	1
TRAJ39	2	4	8
TRAJ40	2	5	1
TRAJ41	3	14	7
TRAJ42	2	7	7
TRAJ43	8	9	4
TRAJ44	1	2	3

TRAJ45	6	13	1
TRAJ46	0	0	0
TRAJ47	0	82	1
TRAJ48	1	30	3
TRAJ49	45	28	17
TRAJ50	1	3	0
TRAJ52	7	5	5
TRAJ53	7	2	6
TRAJ54	1	0	5
TRAJ56	1	0	1
TRAJ57	171	3	0

*Zero represents the corresponding TRDV1-TRAJ pair is not detected.

1. Freeman JD, Warren RL, Webb JR, Nelson BH, Holt RA. Profiling the T-cell receptor beta-chain repertoire by massively parallel sequencing. Genome Res 2009, 19(10): 1817-1824.

2. Warren RL, Freeman JD, Zeng T, Choe G, Munro S, Moore R, et al. Exhaustive T-cell repertoire sequencing of human peripheral blood samples reveals signatures of antigen selection and a directly measured repertoire size of at least 1 million clonotypes. *Genome Res* 2011, **21**(5)**:** 790-797.
